# Crystal structure and Hirshfeld surface analysis of *trans*-2,5-di­methyl­piperazine-1,4-diium tetra­chlorido­cobaltate(II)

**DOI:** 10.1107/S2056989021002954

**Published:** 2021-03-26

**Authors:** Meriem Landolsi, Sonia Abid

**Affiliations:** a Université de Carthage, Faculté des Sciences de Bizerte, LR13ES08, Laboratoire de Chimie des Matériaux, 7021, Zarzouna Bizerte, Tunisia

**Keywords:** crystal structure, tetra­chlorido­cobaltate(II) salt, Hirshfeld surface analysis

## Abstract

In the title mol­ecular salt, (C_6_H_16_N_2_)[CoCl_4_], the complete dication is generated by crystallographic inversion symmetry and the piperazine ring adopts a chair conformation with the pendant methyl groups in equatorial orientations. The complete dianion is generated by crystallographic twofold symmetry. In the crystal, the (C_6_H_16_N_2_)^2+^ and [CoCl_4_]^2−^ ions are linked by N—H⋯Cl and C—H⋯Cl hydrogen bonds, thereby forming a two-dimensional supra­molecular network. The Hirshfeld surface analysis and fingerprint plots reveal that the largest contributions to the crystal stability come from H⋯Cl/Cl⋯H (68.4%) and H⋯H (27.4%) contacts.

## Abstract 2

In the title mol­ecular salt, (C_6_H_16_N_2_)[CoCl_4_], the complete dication is generated by crystallographic inversion symmetry and the piperazine ring adopts a chair conformation with the pendant methyl groups in equatorial orientations. The complete dianion is generated by crystallographic twofold symmetry. In the crystal, the (C_6_H_16_N_2_)^2+^ and [CoCl_4_]^2−^ ions are linked by N—H⋯Cl and C—H⋯Cl hydrogen bonds, thereby forming a two-dimensional supra­molecular network.

## Chemical context   

Tetra­chloro­cobalt/copper (II) salts with organic cations, such as (C_6_H_10_N_3_)_2_[CoCl_4_] (Titi *et al.* 2020 ▸), [(CH_3_)_2_NH_2_]_2_[CoCl_4_] (Pietraszko *et al.* 2006 ▸) and (C_7_H_7_N_2_S)_2_[CuCl_4_] (Vishwakarma *et al.* 2017 ▸) have received attention due to their potential applications in the electronic, magnetic, optical and anti­microbial fields. In these materials, the negative charge on the inorganic complex ion is balanced by the organic groups, which usually act as structure-directing agents by the formation of N—H⋯Cl hydrogen bonds and significantly affect the structure and dimensionality of the supra­molecular network.
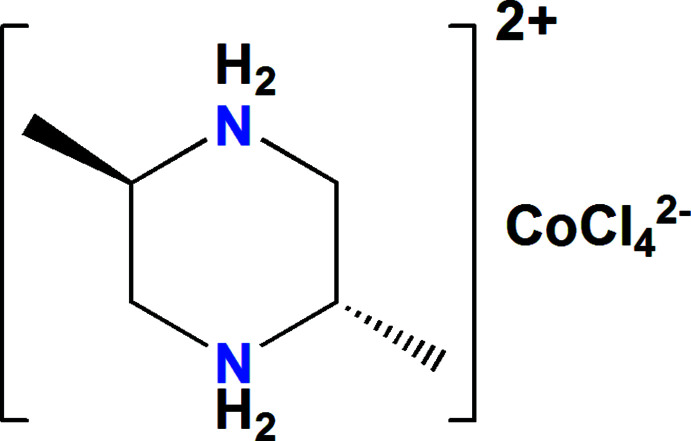


As an extension of these studies, we now describe the synthesis, structure and Hirshfeld surface analysis of the title mol­ecular salt, (I).

## Structural commentary   

**Figure 1:**
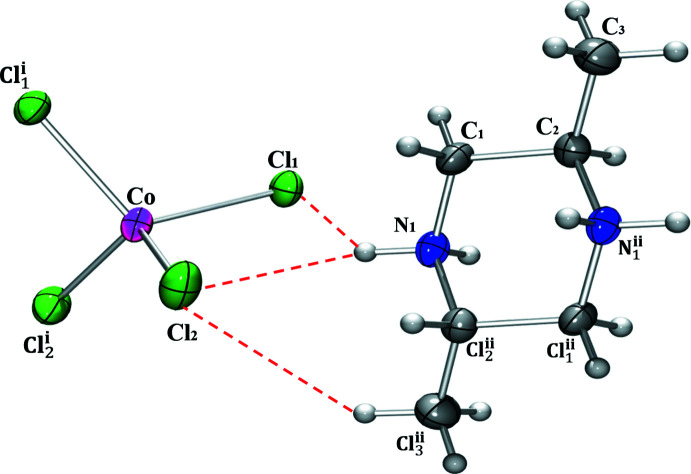
The mol­ecular structure of (I) with displacement ellipsoids set to 50% probability and hydrogen bonds shown as dashed lines. Symmetry codes: (i) −*x* + 1, *y*, −*z* + 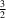; (ii) −*x* + 2, −*y* + 1, −*z* + 1.

The asymmetric unit of (I) comprises half of a *trans*-2,5-di­methyl­piperazine-1,4-dium cation and a half tetra­chlorido­cobaltate anion (Fig. 1 ▸). The cation and anion are completed by crystallographic inversion and twofold symmetry, respectively. In the organic species, the N—C and C—C bond lengths vary from 1.490 (2) to 1.513 (2) Å and the angles C—C—C, N—C—C and C—N—C range from 109.15 (14) to 113.54 (15)°. These data are in agreement with those reported in other salts of the *trans*-2,5-di­methyl­piperazine-1,4-diium cation (Gatfaoui *et al.*, 2014 ▸; Ben Mleh *et al.*, 2016 ▸). The Co^2+^ ion in (I) has a tetra­hedral geometry, with Cl—Co—Cl angles ranging from 103.32 (2) to 116.57 (3)°. The average length of the Co—Cl bonds, 2.27 Å, is close to that observed in similar complexes (Tahenti *et al.*, 2020 ▸; Zhang *et al.*, 2005 ▸; Zeller *et al.*, 2005 ▸).

## Supra­molecular features   

**Figure 2:**
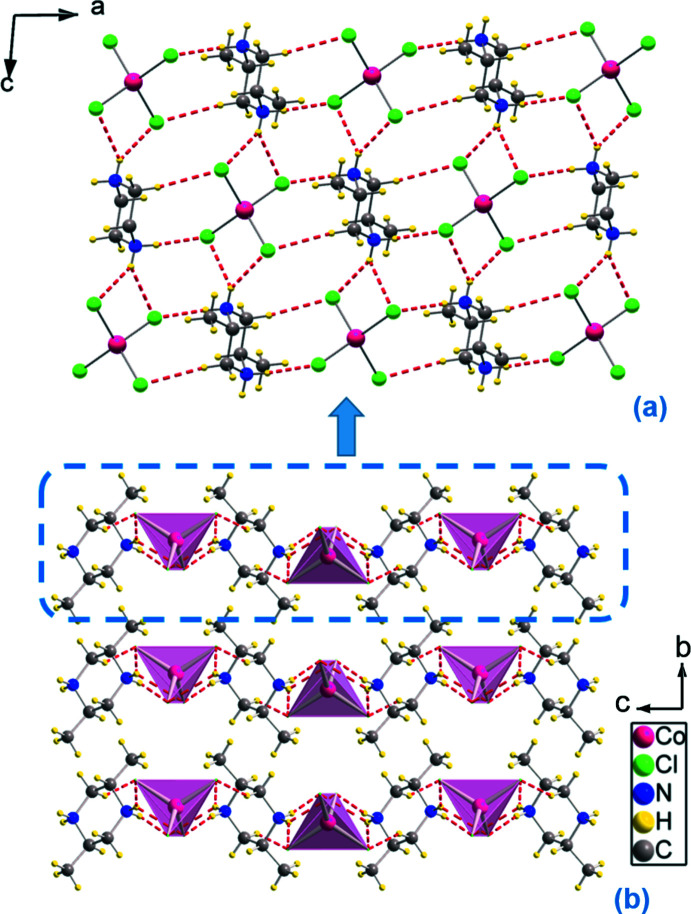
(*a*) Crystal packing in the structure of (I) along the crystallographic *a* axis. (*b*) View of a supra­molecular layer along the *b-*axis direction.

**Figure 3:**
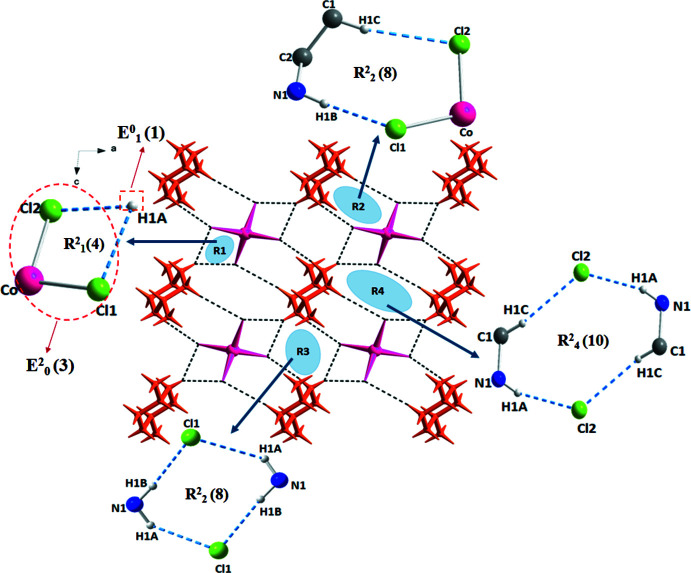
Hydrogen-bonding inter­actions between cations and anions showing the ring patterns of weak inter­actions formed by N—H⋯Cl/C—H⋯Cl links.

In the crystal of (I), adjacent anions are inter­connected by the cations *via* N—H⋯Cl hydrogen bonds and C—H⋯Cl inter­actions (Table 1 ▸) to form a layer built up from the organic and inorganic species, lying parallel to (101) (Fig. 2 ▸). The hydrogen bonds engage the chloride ions of the [CoCl_4_]^2–^ tetra­hedron, producing four types of graph-set motifs on the basis of Etter’s notation (Etter *et al.*, 1990 ▸; Bernstein *et al.*, 1995 ▸). The isolated mol­ecules can be described by the elementary graph-set descriptors *E^a^_d_* (*n*) (Daszkiewicz, 2012 ▸). The graph-set descriptor of the pattern can be easily obtained by the summation of elementary *E^a^_d_* (*n*) graph-sets of isolated ions and mol­ecules. In the case of (I), the elementary graph-sets can be collected (Fig. 3 ▸) as follows:

*E* ^0^_1_ (1) + *E* ^2^_0_ (3) = 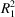 (4)

2*E* ^0^_2_ (3) + 2*E* ^1^_0_ (1) = 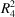 (8)

*E* ^0^_2_ (3) + *E* ^2^_0_ (5) = 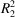 (8)

2*E* ^1^_0_ (1) + 2*E* ^0^_2_ (4) = 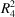 (10).

## Hirshfeld surface analysis   

**Figure 4:**
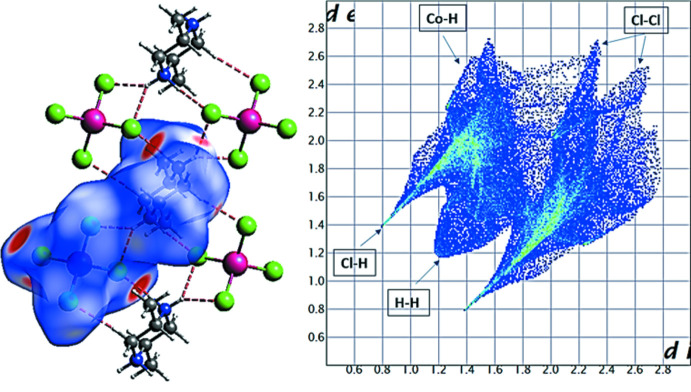
Hirshfeld surface of (I) mapped over *d*_norm_ and the two-dimensional fingerprint plot for all inter­actions.

To further understand the different inter­actions and contacts in the crystal of (I), its Hirshfeld surface (HS) (McKinnon *et al.*, 2004 ▸) was calculated. The *d*_norm_ surface (Fig. 4 ▸) and the associated two-dimensional fingerprint plots (see supporting information) were calculated using *CrystalExplorer 3.1* (Wolff *et al.*, 2013 ▸; Spackman & Jayatilaka, 2009 ▸). This figure shows the areas mapped in the range from −0.480 to 1.048 of the asymmetric ion-pair surrounded by neighboring ions where we can see some of the closest inter­molecular contacts. The large dark-red spots on the HS indicate close contact inter­actions, which are primarily responsible for significant hydrogen-bond contacts. The fingerprint plots indicate that the most important inter­actions are H⋯Cl/Cl⋯H, which cover a HS range of 68.4% and appear as two shape-symmetric spikes in the two-dimensional fingerprint maps (where *d*_i_ ∼*d*_e_ ∼1.4 Å). It should be also noted that the the van der Waals radii of the hydrogen and chlorine atoms are 1.20 and 1.75 Å, respectively. The H⋯H contacts represent the second most abundant inter­actions with 27.4% of the total Hirshfeld surface, including a short H⋯H contact near 2.4 Å (where *d*_i_ ∼*d*_e_ ∼1.2 Å), represented by a cluster of points accumulated on the diagonal of the graph. Other contacts including Cl⋯Cl and Co⋯H/H⋯Co have negligible contributions (respectively 2.7% and 1.5%). It can be concluded that the Cl⋯H/H⋯Cl inter­actions dominate in the title compound.

### Synthesis and crystallization   

A 1:1 mixture of *trans*-2,5-di­methyl­piperazine and cobalt(II) chloride hexa­hydrate was dissolved in a solution of concentrated hydro­chloric acid and the resulting solution was magnetically stirred for 1 h. After two weeks of evaporation, dark-blue prismatic crystals of (I) had formed, which were recovered by filtration and dried in air.

## Refinement   

Crystal data, data collection and structure refinement details are summarized in Table 2 ▸. The N-bound and C-bound hydrogen atoms were positioned geometrically and treated as riding atoms: N—H = 0.86 Å, C—H = 0.96 Å with *U*_iso_(H) = 1.2*U*_eq_(N,C).

## Supplementary Material

Crystal structure: contains datablock(s) I. DOI: 10.1107/S2056989021002954/hb7971sup1.cif

Two-dimensional fingerprint plots. DOI: 10.1107/S2056989021002954/hb7971sup3.pdf

CCDC reference: 1831453

Additional supporting information:  crystallographic information; 3D view; checkCIF report

## Figures and Tables

**Table 1:** Hydrogen-bond geometry (Å, °)

*D*—H⋯*A*	*D*—H	H⋯*A*	*D*⋯*A*	*D*—H⋯*A*
N1—H1*A*⋯Cl1^i^	0.89	2.30	3.1777 (2)	171
N1—H1*B*⋯Cl1	0.89	2.65	3.2594 (2)	126
N1—H1*B*⋯Cl2	0.89	2.49	3.2631 (2)	145
C1—H1*C*⋯Cl2^ii^	0.97	2.82	3.7065 (2)	153

Symmetry codes: (i) -x+2, y, -z+{\script{3\over 2}}; (ii) -x+1, -y+1, -z+1.

**Table 2:** Experimental details

Crystal data	
Chemical formula	C_6_H_16_N_2_^2+^·Cl_4_Co^2−^
*M*_r_	316.94
Crystal system, space group	Monoclinic, *C*2/*c*
Temperature (K)	293
*a*, *b*, *c* (Å)	7.6431 (3), 11.9347 (6), 14.0058 (7)
β (°)	95.519 (4)
*V* (Å^3^)	1271.66 (10)
*Z*	4
Radiation type	Mo *K*α
μ (mm^−1^)	2.15
Crystal size (mm)	0.15 × 0.10 × 0.08

Data collection	
Diffractometer	Agilent SuperNova, Single source at offset, Eos
Absorption correction	Multi-scan (*CrysAlis PRO*; Agilent 2014 ▸)
*T*_min_, *T*_max_	0.816, 1.000
No. of measured, independent and observed [*I* > 2σ(*I*)] reflections	4627, 1546, 1370
*R*_int_	0.029
(sin θ/λ)_max_ (Å^−1^)	0.685

Refinement	
*R*[*F* ^2^ > 2σ(*F* ^2^)], *wR*(*F* ^2^), *S*	0.028, 0.074, 1.08
No. of reflections	1546
No. of parameters	60
H-atom treatment	H-atom parameters constrained
Δρ_max_, Δρ_min_ (e Å^−3^)	0.27, −0.59

Computer programs: *CrysAlis PRO* (Agilent, 2014 ▸), *SIR92* (Altomare *et al.*, 1994 ▸), *SHELXL2017/1* (Sheldrick, 2015 ▸), *DIAMOND* (Brandenburg, 2006 ▸) and *ORTEP*-III (Burnett & Johnson, 1996 ▸).

## supplementary crystallographic information

### Crystal data

**Table:**

C_6_H_16_N_2_^2+^·Cl_4_Co^2^^−^	*F*(000) = 644
*M**_r_* = 316.94	*D*_x_ = 1.655 Mg m^−^^3^
Monoclinic, *C*2/*c*	Mo *K*α radiation, λ = 0.71073 Å
*a* = 7.6431 (3) Å	Cell parameters from 2258 reflections
*b* = 11.9347 (6) Å	θ = 4.1–29.0°
*c* = 14.0058 (7) Å	µ = 2.15 mm^−^^1^
β = 95.519 (4)°	*T* = 293 K
*V* = 1271.66 (10) Å^3^	Prism, blue
*Z* = 4	0.15 × 0.10 × 0.08 mm

### Data collection

**Table:**

Agilent SuperNova, Single source at offset, Eos diffractometer	1370 reflections with *I* > 2σ(*I*)
Detector resolution: 16.0233 pixels mm^-1^	*R*_int_ = 0.029
ω scans	θ_max_ = 29.1°, θ_min_ = 3.4°
Absorption correction: multi-scan (CrysAlisPro; Agilent 2014)	*h* = −10→9
*T*_min_ = 0.816, *T*_max_ = 1.000	*k* = −15→15
4627 measured reflections	*l* = −18→13
1546 independent reflections	

### Refinement

**Table:**

Refinement on *F*^2^	0 restraints
Least-squares matrix: full	Hydrogen site location: inferred from neighbouring sites
*R*[*F*^2^ > 2σ(*F*^2^)] = 0.028	H-atom parameters constrained
*wR*(*F*^2^) = 0.074	*w* = 1/[σ^2^(*F*_o_^2^) + (0.0365*P*)^2^ + 0.515*P*] where *P* = (*F*_o_^2^ + 2*F*_c_^2^)/3
*S* = 1.08	(Δ/σ)_max_ = 0.027
1546 reflections	Δρ_max_ = 0.27 e Å^−^^3^
60 parameters	Δρ_min_ = −0.59 e Å^−^^3^

### Special details

**Table:**

Geometry. All esds (except the esd in the dihedral angle between two l.s. planes) are estimated using the full covariance matrix. The cell esds are taken into account individually in the estimation of esds in distances, angles and torsion angles; correlations between esds in cell parameters are only used when they are defined by crystal symmetry. An approximate (isotropic) treatment of cell esds is used for estimating esds involving l.s. planes.

### Fractional atomic coordinates and isotropic or equivalent isotropic displacement parameters (Å^2^)

**Table:**

	*x*	*y*	*z*	*U*_iso_*/*U*_eq_	
Co1	0.500000	0.52995 (3)	0.750000	0.02694 (13)	
Cl1	0.74705 (6)	0.42227 (4)	0.77647 (3)	0.03237 (14)	
Cl2	0.54849 (7)	0.62944 (5)	0.61790 (4)	0.04332 (16)	
N1	0.9250 (2)	0.51194 (13)	0.58839 (11)	0.0276 (3)	
H1A	1.015221	0.493869	0.630538	0.033*	
H1B	0.833857	0.527625	0.621121	0.033*	
C1	0.8798 (2)	0.41357 (16)	0.52524 (14)	0.0290 (4)	
H1C	0.774246	0.429831	0.483440	0.035*	
H1D	0.856092	0.349324	0.564406	0.035*	
C2	1.0282 (2)	0.38575 (15)	0.46508 (13)	0.0272 (4)	
H2	1.130901	0.361989	0.507561	0.033*	
C3	0.9775 (3)	0.29268 (17)	0.39440 (16)	0.0404 (5)	
H3A	0.947344	0.226813	0.428597	0.061*	
H3B	0.878228	0.315843	0.351734	0.061*	
H3C	1.074621	0.276353	0.358031	0.061*	

### Atomic displacement parameters (Å^2^)

**Table:**

	*U*^11^	*U*^22^	*U*^33^	*U*^12^	*U*^13^	*U*^23^
Co1	0.02007 (18)	0.0374 (2)	0.0236 (2)	0.000	0.00304 (14)	0.000
Cl1	0.0239 (2)	0.0401 (3)	0.0327 (3)	0.00345 (18)	0.00040 (18)	0.00462 (18)
Cl2	0.0334 (3)	0.0564 (3)	0.0408 (3)	0.0047 (2)	0.0073 (2)	0.0198 (2)
N1	0.0252 (7)	0.0368 (8)	0.0217 (8)	−0.0012 (6)	0.0072 (6)	0.0004 (6)
C1	0.0262 (9)	0.0337 (10)	0.0279 (10)	−0.0075 (7)	0.0065 (7)	−0.0013 (7)
C2	0.0253 (8)	0.0307 (9)	0.0255 (9)	0.0012 (7)	0.0023 (7)	0.0026 (7)
C3	0.0450 (11)	0.0375 (11)	0.0394 (12)	−0.0026 (9)	0.0074 (10)	−0.0076 (9)

### Geometric parameters (Å, º)

**Table:**

Co1—Cl2^i^	2.2588 (5)	C1—C2	1.513 (2)
Co1—Cl2	2.2588 (5)	C1—H1C	0.9700
Co1—Cl1	2.2846 (5)	C1—H1D	0.9700
Co1—Cl1^i^	2.2847 (5)	C2—C3	1.513 (3)
N1—C1	1.490 (2)	C2—H2	0.9800
N1—C2^ii^	1.494 (2)	C3—H3A	0.9600
N1—H1A	0.8900	C3—H3B	0.9600
N1—H1B	0.8900	C3—H3C	0.9600
			
Cl2^i^—Co1—Cl2	116.57 (3)	N1—C1—H1D	109.4
Cl2^i^—Co1—Cl1	111.157 (19)	C2—C1—H1D	109.4
Cl2—Co1—Cl1	103.324 (18)	H1C—C1—H1D	108.0
Cl2^i^—Co1—Cl1^i^	103.325 (18)	N1^ii^—C2—C1	109.15 (14)
Cl2—Co1—Cl1^i^	111.155 (19)	N1^ii^—C2—C3	109.31 (16)
Cl1—Co1—Cl1^i^	111.54 (3)	C1—C2—C3	111.50 (16)
C1—N1—C2^ii^	113.54 (15)	N1^ii^—C2—H2	108.9
C1—N1—H1A	108.9	C1—C2—H2	108.9
C2^ii^—N1—H1A	108.9	C3—C2—H2	108.9
C1—N1—H1B	108.9	C2—C3—H3A	109.5
C2^ii^—N1—H1B	108.9	C2—C3—H3B	109.5
H1A—N1—H1B	107.7	H3A—C3—H3B	109.5
N1—C1—C2	111.10 (14)	C2—C3—H3C	109.5
N1—C1—H1C	109.4	H3A—C3—H3C	109.5
C2—C1—H1C	109.4	H3B—C3—H3C	109.5
			
C2^ii^—N1—C1—C2	56.5 (2)	N1—C1—C2—C3	−174.91 (16)
N1—C1—C2—N1^ii^	−54.0 (2)		

Symmetry codes: (i) −*x*+1, *y*, −*z*+3/2; (ii) −*x*+2, −*y*+1, −*z*+1.

### Hydrogen-bond geometry (Å, º)

**Table:**

*D*—H···*A*	*D*—H	H···*A*	*D*···*A*	*D*—H···*A*
N1—H1*A*···Cl1^iii^	0.89	2.30	3.1777 (2)	171
N1—H1*B*···Cl1	0.89	2.65	3.2594 (2)	126
N1—H1*B*···Cl2	0.89	2.49	3.2631 (2)	145
C1—H1*C*···Cl2^iv^	0.97	2.82	3.7065 (2)	153

Symmetry codes: (iii) −*x*+2, *y*, −*z*+3/2; (iv) −*x*+1, −*y*+1, −*z*+1.
