# Supplementary figures and images for: Crystal structure and Hirshfeld surface analysis of trans-2,5-di­methyl­piperazine-1,4-diium tetra­chlorido­cobaltate(II)
Source: Acta Crystallogr E Crystallogr Commun. 2021 Mar 26;77(Pt 4):424–7. doi: 10.1107/S2056989021002954 (PMC8025852; doi:10.1107/S2056989021002954)

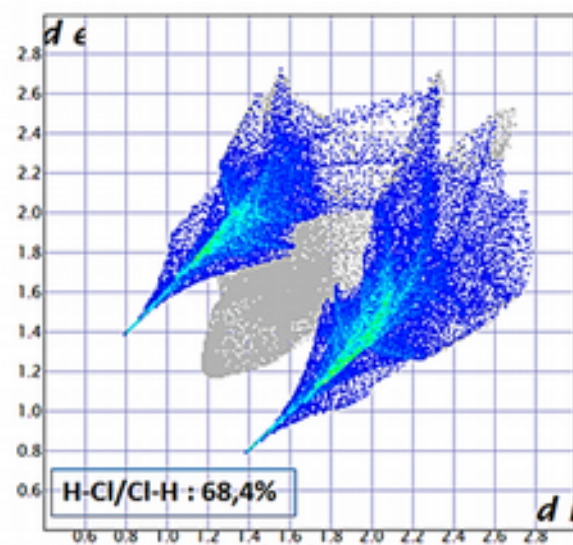

(a)

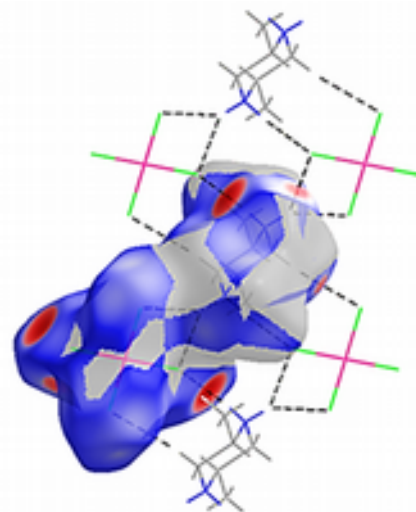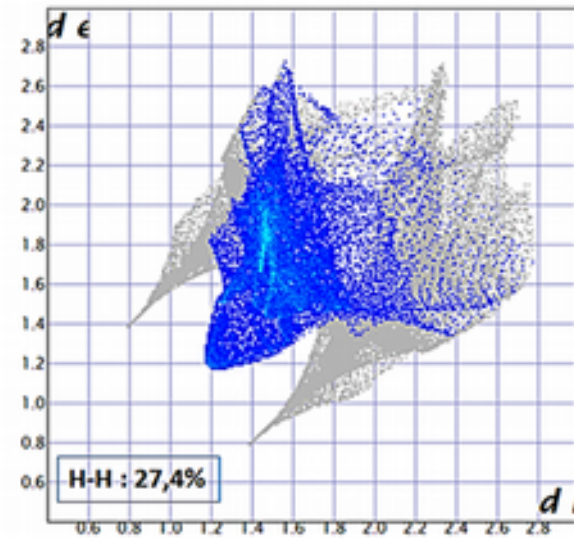

(b)

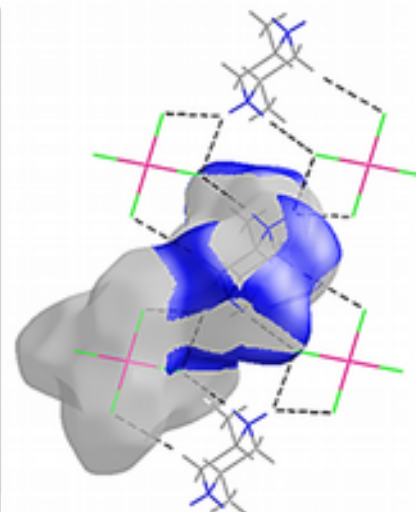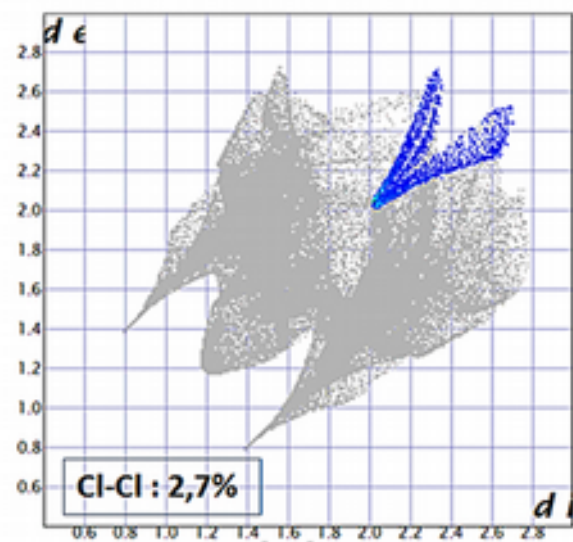

(c)

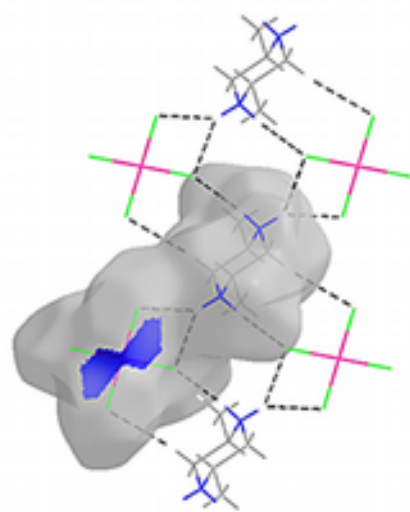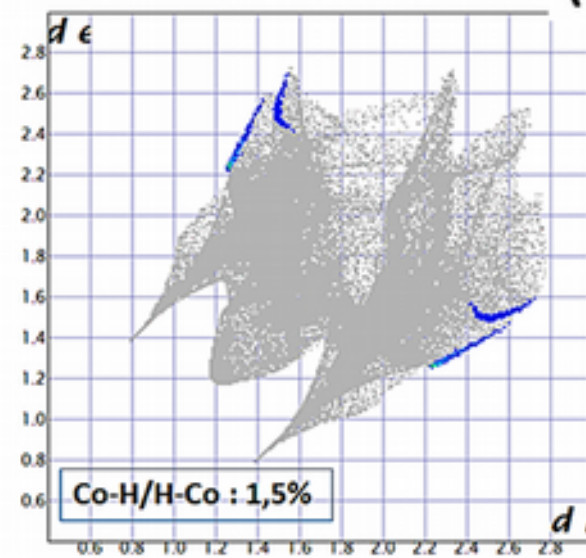

(d)

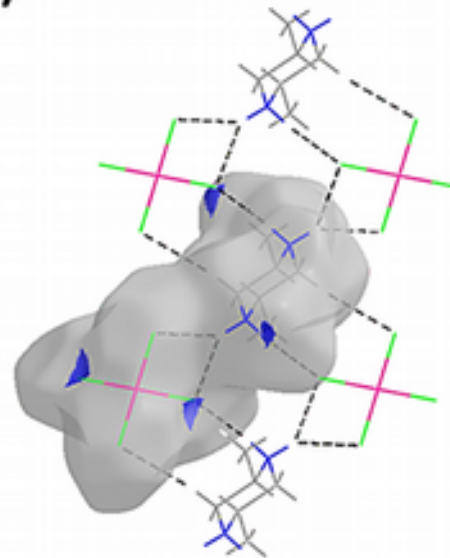

Supplement: Supplementary file 2 [file e-77-00424-sup3.pdf]
